# A case of hyperkeratotic crusted scabies

**DOI:** 10.1371/journal.pntd.0007918

**Published:** 2020-03-05

**Authors:** Adrian Cuellar-Barboza, Jesus A. Cardenas-de la Garza, José A. García-Lozano, Adrian Martinez-Moreno, Gildardo Jaramillo-Moreno, Jorge Ocampo-Candiani

**Affiliations:** Universidad Autónoma de Nuevo León, Facultad de Medicina y Hospital Universitario “Dr. José Eleuterio González”, Servicio de Dermatología, Monterrey, Mexico; Hitit University, Faculty of Medicine, TURKEY

## Abstract

Patients who are immunocompromised or have cognitive or physical disabilities are at a higher risk of being affected with infections such as crusted scabies. This is a rare skin hyperinfestation by *Sarcoptes scabiei* var. *hominis*. The main characteristic of this dermatosis is a thick crust due to the high concentration of mites; in addition, other manifestations such as papules, excoriations, and burrows may be absent. In severe cases, thick yellow-brown crusts and plaques with deep fissures are present. Diagnosis can be made by observing mites, ova, or feces from skin scrapings. Multiple therapies can be used in patients with this condition. Management with patient isolation is important to prevent institutional outbreaks. This disease can have high mortality, primarily due to sepsis. Awareness of this condition and its serious consequences is important to reduce its mortality and morbidity.

## Introduction

Crusted scabies (CS) is a severe and highly contagious variant of the infestation by the *Sarcoptes scabiei* mite. It is characterized by widespread erythematous plaques with prominent scale due to proliferation of thousands or millions of mites. This form of the disease frequently affects immunocompromised, disabled, or debilitated patients [1]. Prompt diagnosis is crucial as patients may develop secondary impetigo, cellulitis, and even sepsis. Diagnosis may be delayed by atypical or extreme clinical presentations that may resemble other causes of erythroderma that present with exuberant scale and crust [2,3].

## Report of a case

**Fig 1:**
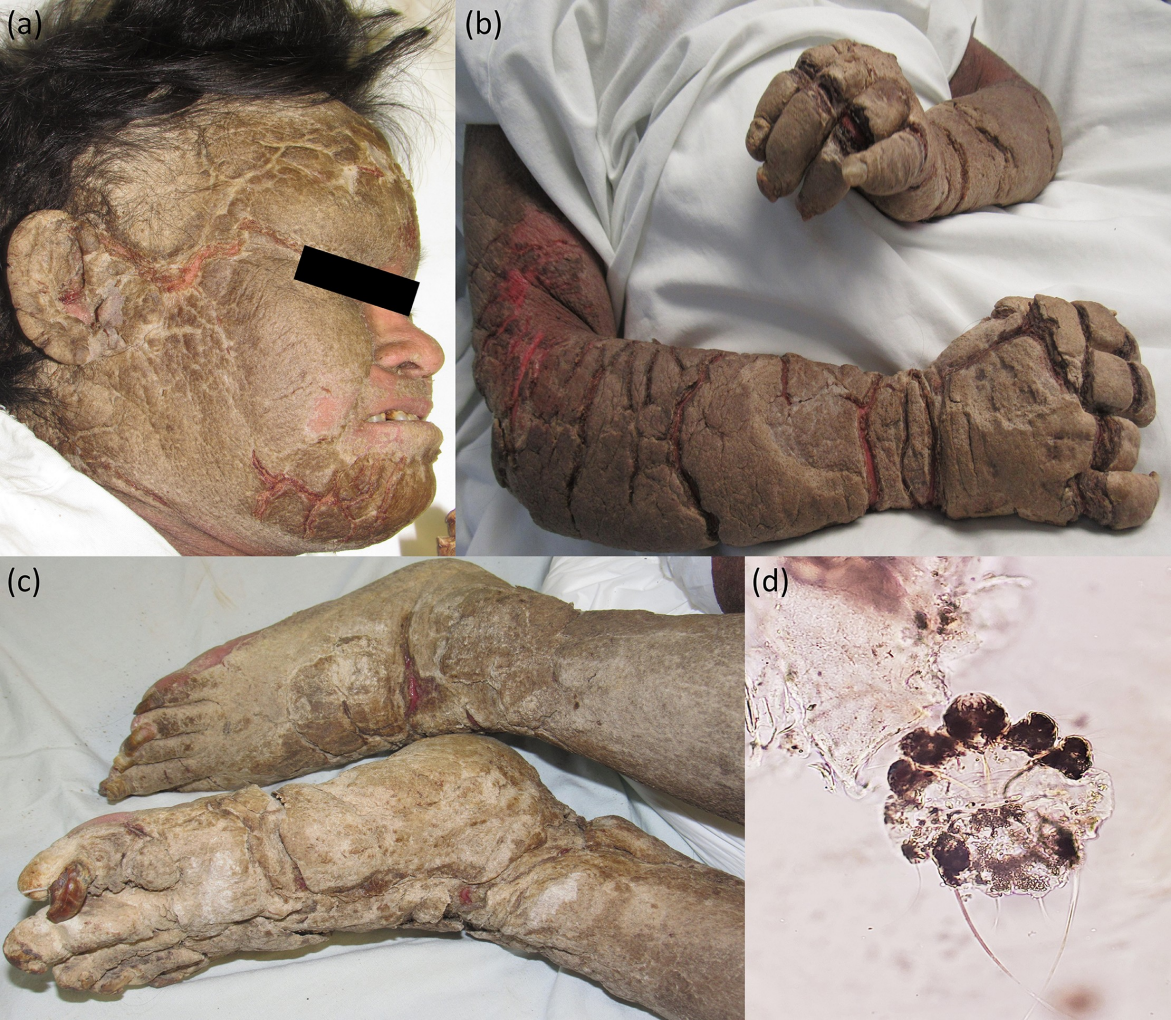
Neglected, hyperkeratotic, and generalized presentation of CS. (A) Face, (B) upper extremities, and (C) lower extremities with thick brown crusts divided by deep fissures with an erythematous background. (D) *S*. *scabiei* mite from a hand skin scraping. Original magnification × 400. CS, crusted scabies.

A 38-year-old woman with Down syndrome was admitted to the emergency unit for food intolerance, malaise, and a generalized chronic dermatosis. She had poor access to sanitation, clean water, or healthcare. Furthermore, she had been mostly confined to a single room for 5 months due to improper care from an elderly, physically disabled family member. There was no personal history of other diseases. Immediately after arrival, the dermatology service was consulted. Her vital signs revealed a blood pressure of 100/60 mm Hg, a heart rate of 90 beats per minute, a respiratory rate of 20 breaths per minute, and an oral temperature of 37.1°C. Oxygen saturation could not be evaluated due to her skin disease. On clinical examination, we identified erythroderma with overlying plaques of prominent, thick scale resembling piled up sand (Fig 1A and Fig 1B). The dermatosis affected the entire body surface, with predominance of the extensor aspect of extremities and the face. Her skin was severely fissured, giving it a rocky appearance (Fig 1C). The keratotic plaques were firm on palpation, and passive movement of her extremities was painful. It was unclear if she experienced pruritus, because she was unable to communicate. Her caretaker had no history of pruritus or a similar dermatosis. Skin scrapings with a No. 15 scalpel dipped in mineral oil were analyzed with direct microscopy, revealing multiple *S*. *scabiei* mites, ova, and feces, confirming the diagnosis of CS (Fig 1D). The patient was then put in isolation to prevent a possible institutional outbreak. Laboratory tests were remarkable for hypernatremia (163 mEq per L; urinary osmolarity of 676 mOsm per kg) and kidney failure with creatinine of 2.7 mg per dL (normal is 0.6–1.4 mg per dL), GFR of 22 mL per min per 1.73 m, and blood urine nitrogen of 24 mg per dl (normal 7–20 mg per dL), attributed to severe dehydration. Intravenous fluids were started, and we initiated oral ivermectin and topical permethrin 5% cream. Unfortunately, briefly after arriving, the patient died. Treatment of her condition was impaired due to the difficulty of accurately measuring vital signs and drawing blood. The presumptive cause of death was septic shock.

## Discussion

CS is a rare skin hyperinfestation by *S*. *scabiei* var. *hominis*. It typically occurs in patients with AIDS, T-cell leukemia, organ transplantation, or diabetes mellitus or patients using systemic steroids [4]. Patients with cognitive or physical disabilities, such as Down syndrome or dementia, also have a higher risk of acquiring this disease [2]. The hallmark clinical sign of this variant of scabies is thick crust. Other classic clinical features such as papules, excoriations, and burrows may be absent. These findings are due to the high concentration of mites that trigger an exaggerated formation of keratin in the stratum corneum [5]. Patients present with erythematous plaques that quickly develop tan crusts that predominate in palms, soles, extensor surfaces, and under fingernails. The diagnosis of atypical cases may be missed for months or years, increasing disease morbidity [4]. In generalized and neglected manifestations, such as our case, clinical diagnosis may be guided by the yellow-to-brown crusts with “piled up sand” appearance affecting the dorsal aspect of nails, fingers, and hands. This thick crust appears in divided segments by deep fissures with an erythematous background that we describe as resembling a “rocky surface.” Diagnosis can be confirmed with a rapid bedside test involving the visualization of mites, ova, or feces from skin scrapings analyzed under direct microscopy. Clinical differential diagnoses include other causes of erythroderma such as psoriasis, ichthyosis, atopic dermatitis, cutaneous lymphoma, pityriasis rubra pilaris, and severe seborrheic dermatitis [3]. To date, treatment guidelines are lacking, but based on case series, the recommended management is a combination of topical and oral agents. Scabicides, such as permethrin 5% cream, and keratolytics (e.g., urea) are efficacious as topical medications. Oral ivermectin is employed in a 5-dose regimen with doses of 200 μg per kg administered on days 1, 2, 8, 9, and 15, with an additional 2 doses given on days 22 and 29 in severe cases [5]. Prophylactic ivermectin (200 μg per kg) for contacts is recommended on days 1 and 14. Furthermore, a key part of management involves reducing the risk of institutional outbreaks by ensuring room isolation, protective garments, cleaning of fomites, and minimizing contact with healthcare staff [5]. In our case, we could not rule out sepsis as a cause of death. Previous studies show an increased mortality rate in patients with CS and *Staphylococcus aureus* bacteremia (SAB) versus patients with only SAB [6].

Scabies is currently considered a neglected tropical disease by the World Health Organization, which brings a spotlight to the disease’s high prevalence in tropical and subtropical regions. Its potential for disfigurement and debilitation can lead to social stigma and the inability to work or attend school [7]. Hence, guidelines are needed to determine specific management protocols to improve outcomes for these patients.

## Ethics statement

Informed written consent was obtained for clinical photographs.

**Learning points**

- CS is a rare skin hyperinfestation by *S*. *scabiei* var. *hominis*.
- Patients with immunosuppression or cognitive or physical disabilities (such as Down syndrome) have a higher risk of acquiring this disease.
- The hyperkeratotic variant of CS is characterized by yellow-to-brown crusts with a “piled up sand” or “rocky” appearance affecting the whole-body surface, including the dorsal aspect of nails, fingers, and hands.
- Mortality rate due to SAB is increased in severe cases of CS.
- Prompt treatment and prevention of institutional outbreaks can improve disease burden.
